# Parachute-Like Mitral Valve Tuberculoma: A Rare Presentation

**DOI:** 10.1155/2017/1023924

**Published:** 2017-10-08

**Authors:** Arslan Masood, Gul Zaman Khan, Irfan Bashir, Zubair Akram

**Affiliations:** Allama Iqbal Medical College, Jinnah Hospital, Lahore, Pakistan

## Abstract

There have been anecdotal reports of tuberculous cardiac involvement, mainly in cases of military tuberculosis or immune deficient individuals. The spectrum of clinical presentations of tuberculous cardiac involvements includes incidental detection of single and multiple well-circumscribed tuberculomas, symptomatic obstructive lesions, AV conduction abnormalities, and even sudden death. We present a case of cardiac tuberculoma in an immune-competent person who presented with worsening dyspnea. The unique morphology of this mass posed an imaging challenge that required 4-dimensional (4D) echocardiography and cardiac magnetic resonance (CMR) detail to differentiate the mass from an anterior mitral leaflet (AML) aneurysm. Histological examination after surgical resection confirmed its tuberculous etiology.

## 1. Introduction

Tuberculosis is one of the leading causes of infectious diseases and death in developing countries [1]. There have been anecdotal reports of tuberculous cardiac involvements including valvular and endocardial lesions, myocarditis, pericarditis, and adjacent extracardiac masses. Tuberculous endocarditis has largely been reported in patients with military tuberculosis or after valve replacement in whom the used valves were already contaminated [2–6]. Here we present a case of cardiac tuberculosis in an immunocompetent patient with isolated cardiac involvement in form of a growth involving mitral valve. Its unique appearance made it an imaging challenge until postoperative histological diagnosis could be made.

## 2. Case Presentation

**Figure 1:**
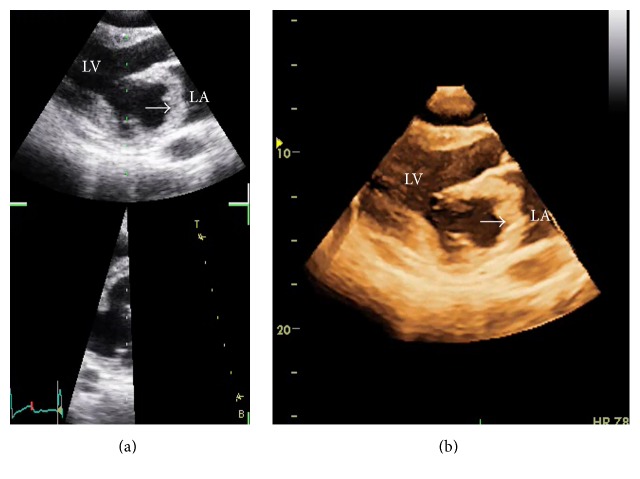
Transthoracic 2D (a) and 4D (b) echocardiographic views in parasternal long axis orientation. Parachute-shaped mass (arrows) in LA attached to atrial side of AML and inferoposterior region of left atrial endocardium. LA: left atrium; LV: left ventricle.

**Figure 2:**
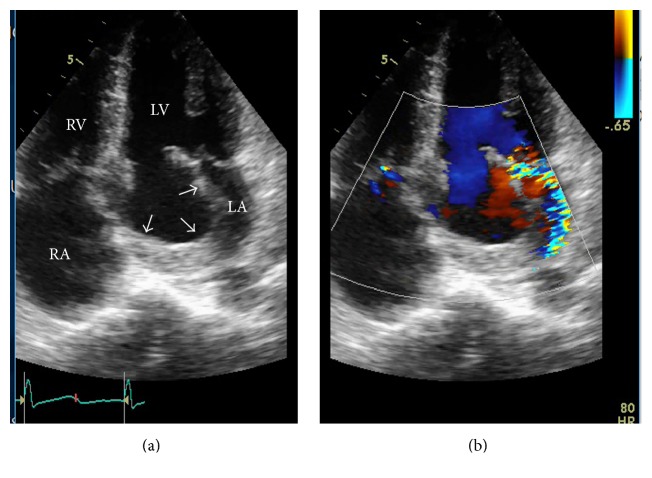
Apical 4-chamber view showing the mass (arrows) mimicking anterior leaflet aneurysm. Color Doppler (b) shows systolic flow into the mass in addition to eccentric MR. LV: left ventricle, LA: left atrium, RV: right ventricle, RA: right atrium, and MR: mitral regurgitation.

**Figure 3:**
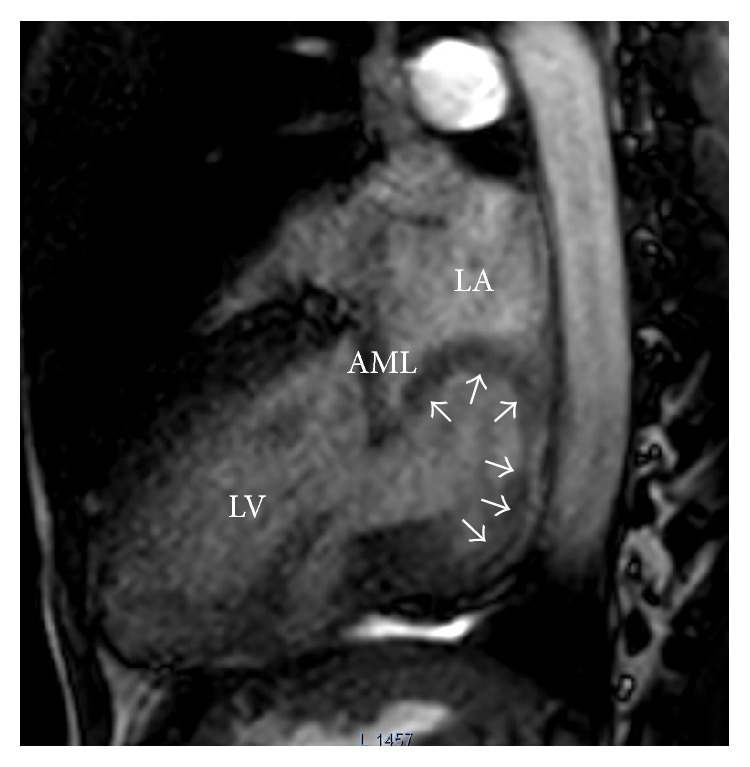
CMR cine run in the sagittal plane reconfirms the thick-walled parachute mass (arrows) tethered to the tip of AML and LA inferoposterior region. Systolic flow into the mass and significant MR are also evident. LA: left atrium, LV: left ventricle, AML: anterior mitral leaflet, and MR: mitral regurgitation.

**Figure 4:**
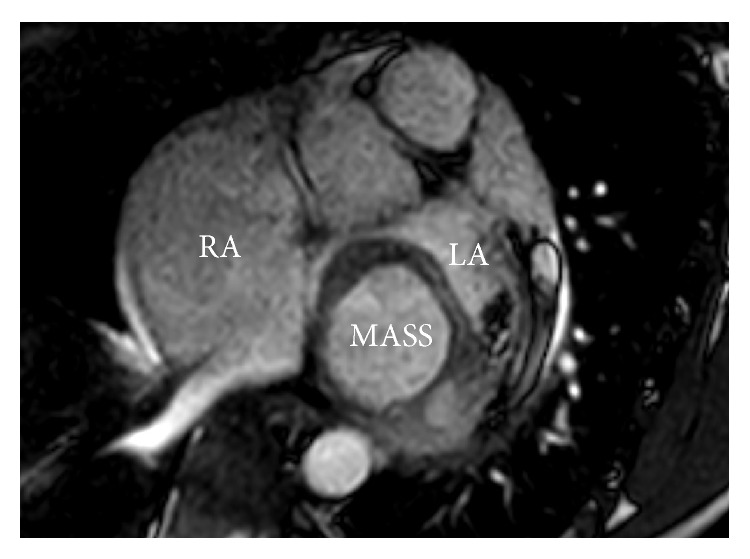
CMR transverse plane image of the LA mass showing its circular shape in cross-section. LA: left atrium; RA: right atrium.

A 38-year-old male presented with worsening dyspnea and gradual weight loss for 9 months. Physical examination revealed a pansystolic apical murmur radiating to axilla with expiratory accentuation. Transthoracic echocardiography revealed a rounded, hollow, parachute-shaped mass in left atrium (LA) near mitral valve leaflets. On initial impression, the mass looked like an aneurysmal deformation of myxomatous AML. However, careful examination on 4D echocardiography delineated the structure as a separate entity from AML. It was hollow, parachute-shaped, mainly tethered to AML and partially to posterior leaflet and adjoining posterior wall of LA (Figure 1). The mass interfered with physiological functioning of mitral valve leading to significant mitral regurgitation (MR). Color Doppler examination revealed a systolic jet both into the cavity of this mass and into the LA (Figure 2). CMR of the structure was carried out to further delineate the mass and assess the possibility of anterior mitral leaflet aneurysm that was ruled out, supporting the 4D echocardiographic morphological description (Figures 3 and 4).

**Figure 5:**
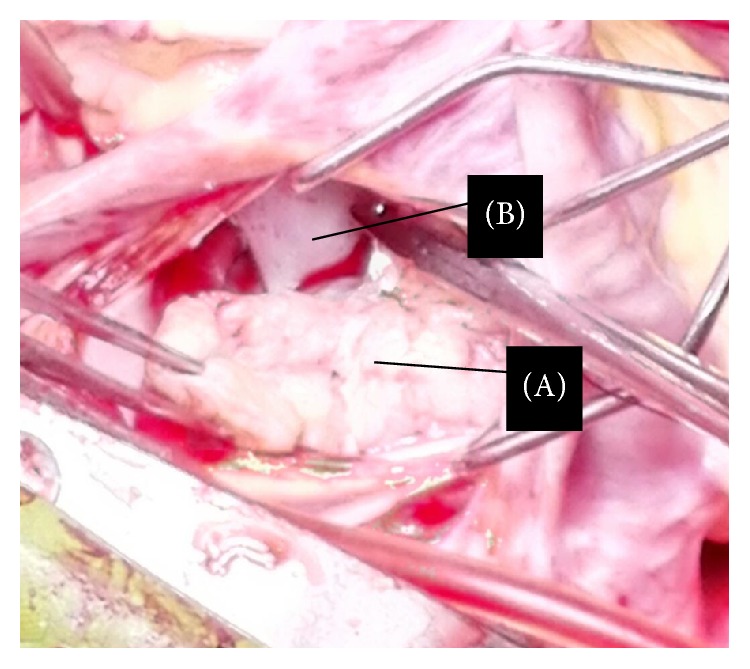
Perioperative image from left atrial access looking at the atrial side of the mass. (A) Parachute-shaped tuberculoma as viewed from above (exposed through LA approach); (B) AML.

**Figure 6:**
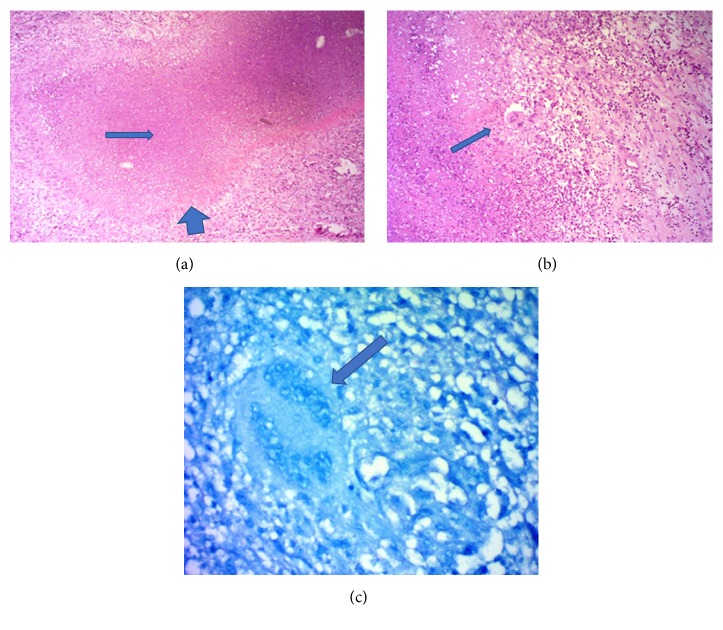
Histological pictures from biopsy specimens of resected mass. (a) Histological appearance of tuberculous granuloma surrounded by palisaded epithelioid histiocytes (broad arrow) with central caseous necrosis (long arrow), (b) magnified histological picture showing a Langhans giant cell (arrow), and (c) Langhans giant cell (arrow) with ZN staining.

Surgical resection of the mass was decided due to significant MR and persistent symptoms. Left atrial approach was taken to expose the mass and its perioperative evaluation was carried out that matched with the consensus morphology on preoperative imaging assessments (Figure 5). Following complete resection of the growth, mitral valve could not be adequately repaired and was replaced by metallic prosthesis. Further postsurgical course was uneventful and a significant improvement in functional capacity was noted. Histological evaluation of tissue sample from the growth revealed areas of caseous necrosis with Langhans giant cells (Figure 6). Standard antituberculous regimen was initiated and continued.

## 3. Discussion

The scientific evidence for cardiac tuberculosis dates back to reports by Maurocordat (1664) and Morgagni (1761) [7]. Postmortem series have documented low frequencies of cardiac tuberculomas in the range of around 0.3% among all tuberculosis patients [8, 9]. Isolated cardiac tuberculomas are even rare [10]. A variety of patterns have been described for cardiac tuberculosis ranging from single to multiple well-circumscribed tuberculomas, most commonly in right sided chambers [11]. There have been sporadic reports of large tuberculous masses including a large right atrial mass leading to tricuspid stenosis in the recent pass [12]. The spectrum of clinical presentations of tuberculous cardiac masses ranges from asymptomatic incidental findings to symptomatic AV conduction abnormalities [10, 13, 14], right ventricular outflow obstruction [15], caval obstructions [2], and even sudden death [16].

Pericardial involvement, the commonest manifest of cardiac tuberculosis in developing nations, is mostly diagnosed by transthoracic echocardiographic findings in the background of clinical picture. Specific histopathological or culture diagnosis is very occasionally required. In contrast, not only are myocardial and endocardial involvements uncommon, but they frequently require histological or cultural evidences in addition to imaging modalities due to lack of specific recognized morphological patterns. Although both histological pattern and Ziehl-Neelsen (ZN) staining are specific for diagnosis, the latter often fails in revealing acid fast bacilli and a definitive diagnosis is made based on typical histological pattern [17].

Given the lack of specific morphological patterns of cardiac tuberculomas, echocardiographic diagnosis is often challenging. The morphological patterns may bear close resemblance to other echogenic intracardiac masses like vegetations, tumors, and aneurysms. A similar diagnostic dilemma was experienced in our case where an initial impression of AML aneurysm needed clarification with 4D and CMR imaging. A conclusive diagnosis could only be made after histological evidence of typical tuberculous morphology following surgical resection. A combination of challenging anatomical detail and rarity of tuberculous etiology made this case unique.

## Supplementary Material

Moving Image -1: Transthoracic 2-D and 4-D echocardiographic views in para-sternal long axis orientation. Parachute-shaped mass can be observed in LA with its open side towards mitral valve and its attachments to atrial side of AML and inferoposterior region of left atrial endocardium. The hollow mass gets inflated in systole due to mitral regurgitation into its cavity.
Moving Image-2: Transthoracic apical 4-chamber view showing the parachute mass in LA with its attachments to AML and LA wall. Color Doppler shows two separate jets of significant MR; one eccentric jet into the true LA cavity along its lateral wall and the other into mass' cavity making it bulge into LA with each systole.
Moving Image-3: CMR in sagittal plane reconfirms the thick-walled parachute mass tethered to the tip of AML and LA inferoposterior region. Systolic flow into the mass and significant MR are also evident.
